# Si-Accumulation In *Artemisia annua* Glandular Trichomes Increases Artemisinin Concentration, but Does Not Interfere In the Impairment of *Toxoplasma gondii* Growth

**DOI:** 10.3389/fpls.2016.01430

**Published:** 2016-09-23

**Authors:** Cristina Rostkowska, Caroline M. Mota, Taísa C. Oliveira, Fernanda M. Santiago, Lilian A. Oliveira, Gaspar H. Korndörfer, Regina M. Q. Lana, Monica L. Rossi, Neusa L. Nogueira, Xavier Simonnet, Tiago W. P. Mineo, Deise A.O. Silva, José R. Mineo

**Affiliations:** ^1^Laboratory of Immunoparasitology, Institute of Biomedical Sciences, Universidade Federal de UberlândiaUberlândia, Brazil; ^2^Fertilizer Technology Laboratory, Institute of Agricultural Sciences, Universidade Federal de UberlândiaUberlândia, Brazil; ^3^Laboratory of Plant Histopathology and Structural Biology of Plants, Center for Nuclear Energy in Agriculture, Universidade de São PauloPiracicaba, Brazil; ^4^Mediplant, Swiss Research Centre on Medicinal and Aromatic PlantsConthey, Switzerland

**Keywords:** *Artemisia annua*, artemisinin, silicon, *Toxoplasma gondii*, herbal medicine

## Abstract

*Artemisia annua* is used as a source of artemisinin, a potent therapeutic agent used for the treatment of infectious diseases, chiefly malaria. However, the low concentration (from 0.01 to 1.4% of dried leaf matter) of artemisinin in the plant obtained with the traditional cropping system makes it a relatively expensive drug, especially in developing countries. Considering that artemisinin and silicon (Si) are both stored in *A. annua* glandular trichomes, and that Si accumulation has never been investigated, this study aimed to look into Si effects on *A. annua* trichome artemisinin concentration, and whether leaf infusion from Si-treated *A. annua* plants is able to control *Toxoplasma gondii* growth. *T. gondii* is the etiologic agent of toxoplasmosis, a zoonotic parasitic disease whose traditional treatment shows significant side effects. The experimental design consisted of *A. annua* seedlings randomly planted in soil treated with different doses of calcium/magnesium silicate (0, 200, 400, 800, and 1600 kg ha^-1^). Analysis of foliar macronutrients showed significant increases of nitrogen content only at the highest dose of silicate. Foliar micronutrients, Si concentrations, and plant height were not affected by any of the silicate doses. However, the dose of 400 kg ha^-1^ of silicate increased the trichome size, which in turn raised artemisinin concentration in leaves and the infusion. In contrast, the 800 and 1600 kg ha^-1^ doses dramatically decreased artemisinin concentration. HeLa cell treatment with the infusion of *A. annua* grown in soil treated with 400 kg ha^-1^ of silicate decreased parasite proliferation in a dose-dependent manner when the treatment was carried out after or along with *T. gondii* infection. However, this effect was similar to *A. annua* grown in soil without silicate treatment. Thus, it can be concluded that, even though Si applied to the soil at 400 kg ha^-1^ has a positive effect on the *A. annua* glandular trichome size and the artemisinin concentration, this outcome cannot be directly associated with the efficiency of *A. annua* infusion on *T. gondii* growth, suggesting that other components from *A. annua* leaves could be acting in synergy with artemisinin.

**GRAPHICAL ABSTRACT:**
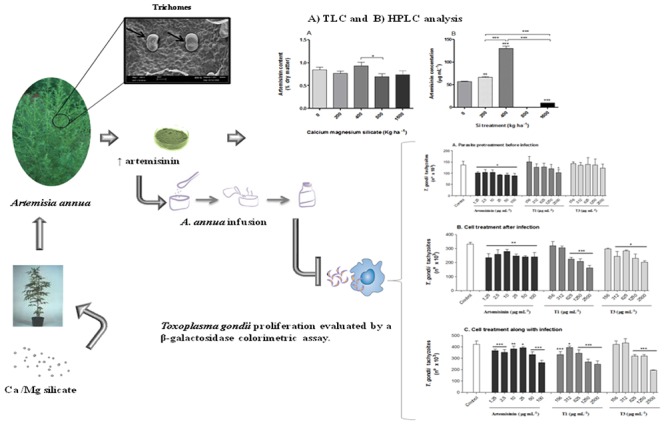
**Summary of the main strategies used and results obtained in the present study**.

## Introduction

Artemisinin is a sesquiterpene lactone endoperoxide, that is produced and stored in glandular trichomes of *Artemisia annua*; which are present especially on the surfaces of leaves and flowers. Trichomes also accumulate elements such as silicon (Si), which is present in cell wall in the form of silica. Therefore, knowing that the trichome is a specific site of artemisinin biosynthesis and sequestration, it may be useful to investigate the effect of Si on trichome density, its morphology and the accumulation of artemisinin in these plant structures, as it has already been analyzed for wheat trichomes (Duke and Paul, 1993; Wagner et al., 2004). *A. annua* infusion containing artemisinin has been used to treat malaria since its discovery in the Chinese herbal garden (Miller and Su, 2011). More recently, *A. annua* and its active components, such as artemisinin, have been used to treat other protozoan infections (Derda et al., 2016; Golami et al., 2016). However, one of the major handicaps is the low concentration of artemisinin in the infusion, justifying the quest for alternative pathways to increase the concentration of this active compound (Bryant et al., 2016; Kiani et al., 2016; Pandey and Pandey-Rai, 2016). In Brazil, studies with *A. annua* started with the initiative of a group of researchers who imported the plant, isolated the active ingredient and worked to obtain hybrid plants able to deliver higher levels of artemisinin (Magalhães et al., 1999).

Silicon is an abundant element in the soil, mainly found as silica and silicate minerals. It is considered a beneficial element, which can improve crop growth, production, and tolerance to biotic and abiotic stress factors (Datnoff et al., 2001; Epstein, 2009). Indeed, Si has been used for centuries to prevent diseases in agriculture. The deposition of Si in the cell wall of wheat leaves and awns is correlated with the localization of silica in the trichomes, giving the leaves their roughness and toughness; acting as a physical barrier by impeding the penetration of the epidermis by herbivores and pathogens (Epstein, 2009). Previous study using scanning electron microscopy (SEM) in addition to X-ray microanalysis clearly demonstrated the deposition of solid, amorphous silica, Si0_2_ – nH_2_0, or “opal phytoliths” in the trichomes of wheat plants (Epstein, 2009). However, to the best of our knowledge, there is no data elucidating the effect of Si on the *A. annua* physiology, particularly considering its effect on the artemisinin content in glandular trichomes.

Toxoplasmosis is caused by *Toxoplasma gondii*, an obligate intracellular protozoan whose life cycle involves *Felidae* definitive hosts and warm-blooded intermediate hosts, including domestic and wild animals, birds, and humans (Tenter et al., 2000). The hosts are infected by ingesting sporulated oocysts with water or food, previously contaminated by infected cats, or by ingesting bradyzoites within tissue cysts in raw or undercooked meat from infected animals. Fetuses can also be infected by vertical transmission of tachyzoites during primary maternal infection. Most infections are asymptomatic in immunocompetent subjects; however, they can be severe in immunocompromised patients, as opportunistic infections cause miscarriages or birth defects in pregnant non-immune animals and humans (Boothroyd and Grigg, 2002; Montoya and Liesenfeld, 2004).

This study aimed to explore the effects of Si on the *A. annua* physiology and artemisinin content, as well as the possibility to control *T. gondii* growth in cell culture with infusion from *A. annua* plants treated with various Si doses. Considering the clinical importance of toxoplasmosis for determined patients at risk, in addition to the substantial side effects of the currently available chemotherapy, which displays high toxicity (Haverkos, 1987; Leport et al., 1988), the effectiveness of artemisinin and its derivatives to control *T. gondii* growth constitutes a new pathway of treatment of *T. gondii* infection, due to its low toxicity and inhibitory effect on the parasite.

## Materials and Methods

### Experimental Site

Seeds of *A. annua* L. were provided by Dr. Pedro Melillo de Magalhães (University of Campinas-UNICAMP, Brazil). The soil was collected from the top 20 cm layer, air dried, sieved through a 5-mm screen and homogenized. The soil was classified as eutroferric red latosol (clayey Oxisol). Soil chemical analysis demonstrated the following results: pH H_2_O 4.4; P 3.4 mg dm^-3^ (extracted with H_2_SO_4_ 0.0125 mol L^-1^ + HCl 0.05 mol L^-1^); Al 1.0 Cmol_c_ dm^-3^; Ca 0.1 Cmol_c_ dm^-3^;H+Al 6,90 Cmol_c_ dm^-3^; Mg 0.1 Cmol_c_ dm^-3^; K 0.17 Cmol_c_ dm^-3^; Sum of bases 0.37 Cmol_c_ dm^-3^; effective CEC 1.37 Cmol_c_ dm^-3^; CEC (pH 7.0) 7.7 Cmol_c_ dm^-3^; base saturation 5.0%; aluminum saturation 73%; and organic matter 33 g kg^-1^. The values for B, Cu, Fe, Mn, and Zn were 0.23, 0.6, 79, 3.3, and 0.6 mg dm^-3^, respectively. Si content in the initial soil was 7.8 mg kg^-1^.

The experimental design consisted of five randomized blocks with five replications. *A. annua* seedlings, each approximately 15 cm tall (4–8 weeks after planting), were transplanted to pots containing 10 kg of soil with different Si doses (an equivalent of 0, 200, 400, 800, and 1600 kg ha^-1^) of calcium and magnesium silicate (MgCaSiO_3_: 25% of Ca, 6% of Mg, and 10.5% of Si). Each pot was fertilized with: 8.33 g of superphosphate; 1.11 g of urea; 2.58 g of KCl; 0.5 g of FTE; and Recmix (Ca and Mg silicate: T1 = 0 g per pot, T2 = 9.52 g per pot, T3 = 19.05 g per pot, T4 = 38.10 g per pot, and T5 = 76.19 g per pot). Fertilizers were incorporated homogeneously to the entire volume of soil and the pots were randomly arranged in a greenhouse (ICIAG, UFU, Brazil). The plants were periodically irrigated. No herbicides or pesticides were applied to the plants.

### Analysis of Macro and Micronutrients, and Si Content In *A. annua*

The analysis of macro and micronutrients in *A. annua* leaves was performed according to the protocol described by Embrapa, Centro Nacional de Pesquisa de Solos (2009). The Si analysis in *A. annua* plants were carried out according to the method described by Elliott and Snyder (1991) and adopted by Korndörfer et al. (2004). Accordingly, leaf samples were weighed (0.1 g) and placed into plastic tubes, then 2 ml of H_2_O_2_ (30 or 50%) and 3 ml of NaOH (1:1) were added. After stirring, the tubes were immediately autoclaved for 1 h at 123°C and 1.5 atm. The digested material was mixed with 2 mL of ammonium molybdate, 1:5 [(NH_4_)_6_ Mo_7_O_24_.4H_2_O: distilled water] forming the yellow complex of silicomolybdic acid [H_4_ (SiMo_12_O_40_)]. Formation of the silicomolybdic acid is maximum between pH 1.0 and 2.0. To lower the pH of the samples, the proportional volume of HCl (50%) was added to the aliquot. The 1-amino-2-naphthol-4-sulfonic acid (reducer) used to eliminate the interference of P and Fe was substituted by 2 mL of oxalic acid [75 g of (COOH)_2_. 2H_2_O in 200 mL of distilled water] per sample. Si concentrations in the extracts were read on a photocolorimeter at 410 nm wavelength. The results of foliar Si in relation to the dry weight of the leaves were expressed as mg kg^-1^ of dry weight of leaves.

### Analysis of Glandular Trichomes In *A. annua* Leaves

As the artemisinin and Si are deposited in glandular trichomes, the experiment aimed to verify the effect of Si treatments on the size and integrity of *A. annua* glandular trichomes. The analysis of the trichomes in leaves was performed according to the method described by Kitajima and Leite (1999). First, a leaf from the middle third of each plant in all treatments was collected in the morning, washed, dried, cut into pieces, and immediately fixed using modified Karnowsky solution (2% glutaraldehyde, 0.05 M sodium cacodylate buffer, pH 7.2; Karnovsky, 1965). The post-fixation was performed with osmium tetroxide in 1% sodium cacodylate buffer 0.05 M, pH 7.2, for 1 h. Then, the samples were immersed three times in distilled water and dehydrated in increasing concentrations of alcohol (30, 50, 70, and 80%). Next, the samples were washed three times with 100% alcohol and dried to the critical point using liquid carbon dioxide. The dried samples were mounted on small metal cylinders and bathed in 20 nm gold in MED 010 evaporator (Balzer Union Ltd, Balzers, Liechtenstein). The images were obtained with Zeiss LEO 435 VP scanning electron microscope (Leo Electron Microscopy Ltd Cooperation Zeiss Leica, Cambridge, England) at 20 kV. Part of this material was covered with carbon for X-ray microanalysis by Zeiss 940 DSM-A scanning microscope (Carl Zeiss Inc, Oberkochen, Germany). The images obtained were used for the measurements of total and intact glandular trichomes with the aid of the ImageJ analysis software, as previously described (NIH, Bethesda, MD, USA; Cornelissen et al., 2003).

### Determination of Artemisinin Content In *A. annua* Leaves

Artemisinin content was determined in *A. annua* leaves by thin layer chromatography (TLC), according to Quennoz et al. (2010). Fresh leaves were collected, dried, ground, and sent to Mediplant, Center for Research on Medicinal Plants, Conthey, Switzerland. Densitometric evaluation was performed with a CAMAG TLC scanner (CAMAG, Muttens, Switzerland). Artemisinin content was expressed in percentage (grams of artemisinin per 100 g of dry weight).

### Preparation of *A. annua* Infusion

The leaves of *A. annua* were harvested before the flowering period for the best artemisinin content. Immediately after the harvest, the leaves were washed with distilled water, dried in a forced circulation oven at 45°C for 24 h, grounded in a Wiley mill, packed in labeled plastic bags and stored at low humidity and temperature. Subsequently, part of the material was subjected to the analysis of macro and micronutrients and the quantification of leaf Si, while the other part was infused and lyophilized for the analysis of artemisinin content.

The *A. annua* infusion was prepared by adding 10 g of dried leaves to 100 mL of boiling distilled water. The mixture was briefly stirred and covered for 10 min. Next, the plant material was removed by filtration and cooled down at room temperature (Räth et al., 2004). The *A. annua* L. infusion (100 mg mL^-1^) was distributed into 1 mL aliquots, lyophilized and stored at 4°C for the analysis by high performance liquid chromatography (HPLC). Part of this stock solution was filtered through syringe filters with 0.2 μm pore size (Millipore Corporation, Bedford, MA, USA) and immediately used in cytotoxicity assays.

### Determination of Artemisinin Content In *A. annua* Infusion

The lyophilized stock solution of *A. annua* infusion was resuspended in 1 mL of acetonitrile (Sigma-Aldrich Corporation, St. Louis, MO, USA) and centrifuged at 13,400 *g* for 5 min. The supernatant was filtered through syringe filters with 0.2 μm pore size (Millipore Corporation, Bedford, MA, USA) prior to HPLC analysis. Artemisinin content was determined using the Shimadzu Prominence SPD-M20A HPLC PDA detector equipped with a UV-vis diode array detectors and LC Solution software (Shimadzu Co., Kyoto, Japan), and based on the standard calibration curve of pure artemisinin (Sigma-Aldrich Corporation, St. Louis, MO, USA) with twofold serial dilutions from 500 to 31.25 μg mL^-1^.

The twofold serial dilutions consisted of a test tube with the stock solution (500 μg of pure artemisinin + 1 mL of acetonitrile) and five other test tubes each containing 500 mL of acetonitrile (solvent). Using a micropipette, 500 mL of the stock solution was transferred to the first tube. After mixing, 500 mL from this tube was transferred to the next one. The same procedure was carried out until the last tube, which eventually contained 31.25 μg mL^-1^. Therefore, the twofold dilution reduced the concentration of the stock solution in the second test tube by a factor of two. In the following test tubes the concentration was further diluted reaching 31.25 μg mL^-1^ in the fifth tube.

The Shim-pack VP-ODS (150 mm × 4.6 mm, 5 μm) reverse phase column (Shimadzu Co, Kyoto, Japan) was used to separate the samples. Total chromatograms were recorded from 0 to 12.5 min by DAD detector system. The chromatographic peak density values were used to estimate the levels of artemisinin in the samples.

### Cell Culture and Cytotoxicity Assays

HeLa cells were obtained from American Type Culture Collection (ATCC, Manassas, VA, USA) and cultured in RPMI-1640 medium supplemented with 25 mM HEPES, 2 mM L-glutamine, 100 U mL^-1^ penicillin, 100 μg mL^-1^ streptomycin (all reagents from Sigma-Aldrich Corporation, St. Louis, MO, USA), and 10% heat-inactivated fetal calf serum (FCS; Cultilab, Campinas, Brazil) in a humidified incubator at 37°C and 5% CO_2_.

The cytotoxicity of *A. annua* infusion samples and pure artemisinin were evaluated for cell viability with a MTT assay (Mosmann, 1983). The MTT cell proliferation assay is a method to determine the cell number using standard microplate absorbance readers. The reduction of tetrazolium salts is a reliable way to examine cell proliferation. It involves the conversion of the water-soluble yellow tetrazolium MTT (3-(4,5-dimethylthiazol-2-yl)-2,5-diphenyltetrazolium bromide) to the insoluble formazan by metabolically active cells. The resulting intracellular purple formazan is then solubilized, and the concentration determined by optical density at 570 nm.

HeLa cells were cultured in the presence of *A. annua* infusion (twofold serial dilutions from 10,000 to 156.25 μg mL^-1^), pure artemisinin (twofold serial dilutions from 400 to 3.125 μg mL^-1^), or medium alone (control). After 24 h of incubation, the cells were washed and incubated for 4 h with 0.5 mg mL^-1^_._ Thiazolyl Blue Tetrazolium Blue (MTT; Sigma-Aldrich Corporation, St Louis, MO, USA). The absorbance of the light emitted by purple formazan was measured at 570 nm using a microtiter plate reader. The results were expressed as percentage of cell viability when compared to controls.

### Parasites

*Toxoplasma gondii* tachyzoites (2F1 RH strain) were propagated in HeLa cells maintained in RPMI-1640 medium supplemented with penicillin, streptomycin and 2% of FCS at 37°C and 5% CO_2_. The *T. gondii* 2F1 strain, which constitutively expresses cytoplasmic β-galactosidase, was provided by Dr. Vern Carruthers (University of Michigan, USA).

### Infusion of *A. annua* on *T. gondii* Infection *In vitro*

In all experimental and infection conditions, a reference curve was prepared with double serial dilutions of *T. gondii* tachyzoites of 2F1 (RH) ranging from 1 × 10^6^ to 1.6 × 10^4^ parasites in 100 μL of a medium with 2% of fetal bovine serum (FBS). After 24 h of incubation at 37°C and 5% CO_2_, the plates were centrifuged for 5 min at 4°C. Next, the supernatant was discarded and the excess of the parasites was removed from the monolayers by washing with cold phosphate buffered saline (PBS, pH 7.2). Then, the cells were lysed with 100 μL of ice-cold lysis buffer (100 mM HEPES, pH 8.0, 1 mM MgSO_4_, 0.1% Triton X-100, 5 mM dithiothreitol) for 15 min. Next, the lysis buffer was removed and 160 μL per well of assay buffer was added (100 mM phosphate buffer, pH 7.3, 102 mM β-mercaptoethanol, and 9 mM MgCl_2_) and 40 μL of 3 mM CPRG chlorophenol red-β-D-galactopyranoside (Roche Diagnostics, Indianapolis, IN, USA). The enzyme activity of β-galactosidase was determined after 30 min at room temperature in the dark at 570 nm in a plate reader (Molecular Devices, Palo Alto, CA, USA). Results were expressed as the number of tachyzoites calculated in relation to the reference curve with 2F1 tachyzoites.

In the first set of the experiment (parasites pre-treated before the infection) 2F1 RH tachyzoites were pre-treated for 1 h at 37°C and 5% of CO_2_ with twofold serial dilutions of *A. annua* infusion (2500–156 μg mL^-1^) obtained from: (i) plants without (0 kg ha^-1^) or with (400 kg ha^-1^) calcium/magnesium silicate applied to the soil, (ii) pure artemisinin (100–1.25 μg mL^-1^), or (iii) medium alone (control). Parasites were then added to HeLa cell monolayers at 3:1 ratio (parasite:cell) and incubated for 24 h at 37°C and 5% of CO_2_. Parasite intracellular proliferation was determined by β-galactosidase colorimetric assay at 570 nm (Jones-Brando et al., 2006).

In the second set of the experiment (cells treated after the infection) HeLa cell monolayers were infected with 2F1 RH tachyzoites at 3:1 (parasite:cell) for 3 h at 37°C and 5% of CO_2_. After washing, infected cells were treated with different concentrations of *A. annua* infusion, artemisinin, or medium alone (control) as described above. Parasite proliferation was determined as described for the first experimental condition.

In the third set of the experiments (cells treated immediately after the infection) HeLa cell monolayers were infected with 2F1 RH tachyzoites at 3:1 (parasite:cell) and then immediately treated with different concentrations of *A. annua* infusion, artemisinin, or medium alone (control) as described above. Parasite proliferation was determined as described for the earlier experiments. Two independent experiments were performed with four replicates for each experimental condition.

### Statistical Analysis

All data were expressed as mean ± mean standard error (MSE). Statistical analysis was performed using the GraphPad Prism version 5.0 software (GraphPad Software, Inc., San Diego, CA, USA). The differences in the total trichome density, percentage of intact trichome, trichome mean size, artemisinin contents, and parasite proliferation were determined by ANOVA and Bonferroni multiple comparison test. Significant differences were considered when *p* < 0.05.

## Results

### Effects of Si Treatment on *A. annua* Nutritional Status

**FIGURE 1:**
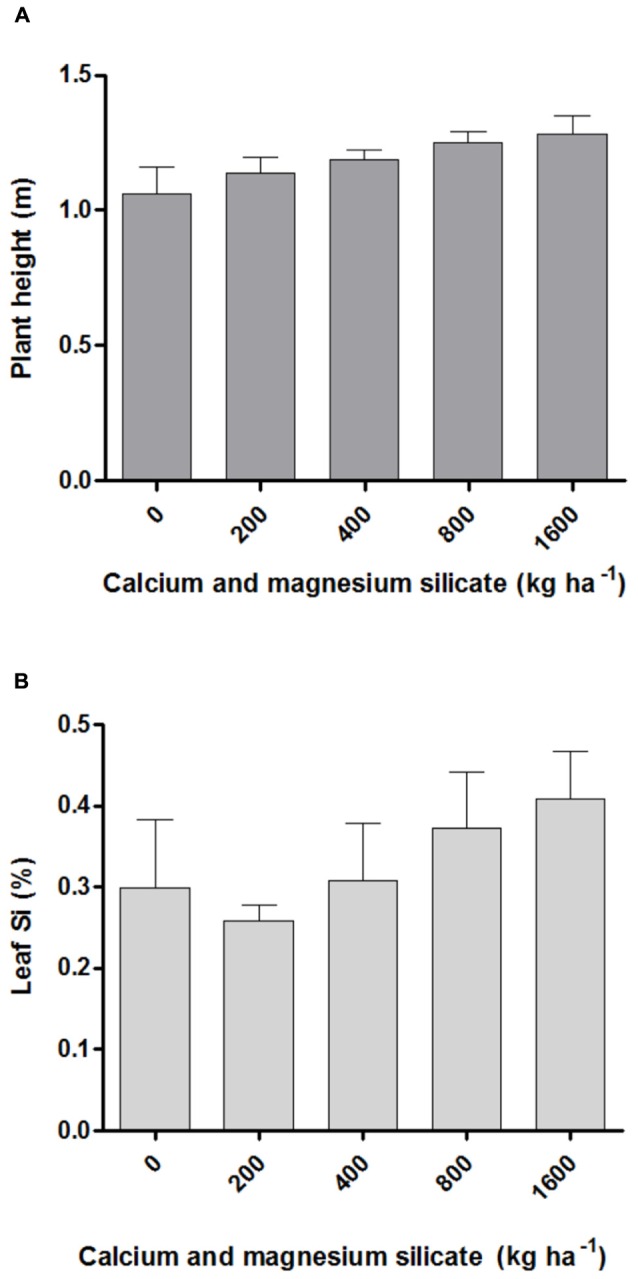
**Plant height (A) and silicon content in leaves (B) of *Artemisia annua* after calcium/magnesium silicate application in the soil.** Data represent mean ± mean standard error (MSE).

The effect of different Si doses applied to the soil on the macronutrient content in *A*. *annua* leaves was significant only for nitrogen, which increased with the highest silicate dosage (1600 kg ha^-1^). The average N content after Si application varied from 19.04 g kg^-1^ for 0 Si kg ha^-1^ to 29.48 g kg^-1^ for the dose of 1600 kg ha^-1^, an increase by 64.6% relative to the control (data not shown). However, the increased N content induced by the highest silicate dose applied to the soil was neither correlated with plant height (**Figure 1A**) nor the artemisinin content (**Tables 1** and **2**). The content of micronutrients was not significantly affected by silicate doses (data not shown). Furthermore, the *A*. *annua* plant exhibited high tolerance to soil acidity, with adequate growth even in conditions of low soil fertility, as determined in its initial chemical characterization. In addition, the different Si doses applied to the soil did not significantly affect the Si content in *A*. *annua* leaves, which values ranged from 0.3 (control) to 0.4% (1600 kg ha^-1^; **Figure 1B**).

**Table 1:** Artemisinin concentrations, analyzed by TLC (thin layer chromatography), in leaves of *Artemisia annua* grown in soil treated with Si at doses of 200, 400, 800, or 1600 kg ha^-1^, in comparison with non-treated soil.

TLC					
	Calcium and Magnesium silicate (kg ha^-^^1^)				
	0	200	400	800	1600
Artemisinin content (μg mL^-1^)	0.7	0.7	1.0	0.6	0.6
	0.8	0.8	1.0	0.6	0.5
	0.9	0.7	1.1	0.8	0.7
	0.8	0.8	0.7	0.6	1.0
	1.0	0.9	0.9	0.9	0.9
**Mean**	**0.8**	**0.8**	**0.9**	**0.7**	**0.7**

**Table 2:** Artemisinin concentrations, analyzed by HPLC (high performance liquid chromatography), in leaves of *A. annua* grown in soil treated with Si at doses of 200, 400, or 1600 kg ha^-1^, in comparison with non-treated soil.

HPLC				
	Calcium and Magnesium silicate (kg ha^-^^1^)			
	0	200	400	1600
Artemisinin content (μg mL**^-^**^1^)	57.4	65.4	133.9	9.8
	56.7	65.5	125.3	10.2
	56.0	68.4	132.3	9.6
**Mean**	**56.7**	**66.5**	**130.5**	**9.9**

### Glandular Trichomes and Artemisinin Concentrations

**FIGURE 2:**
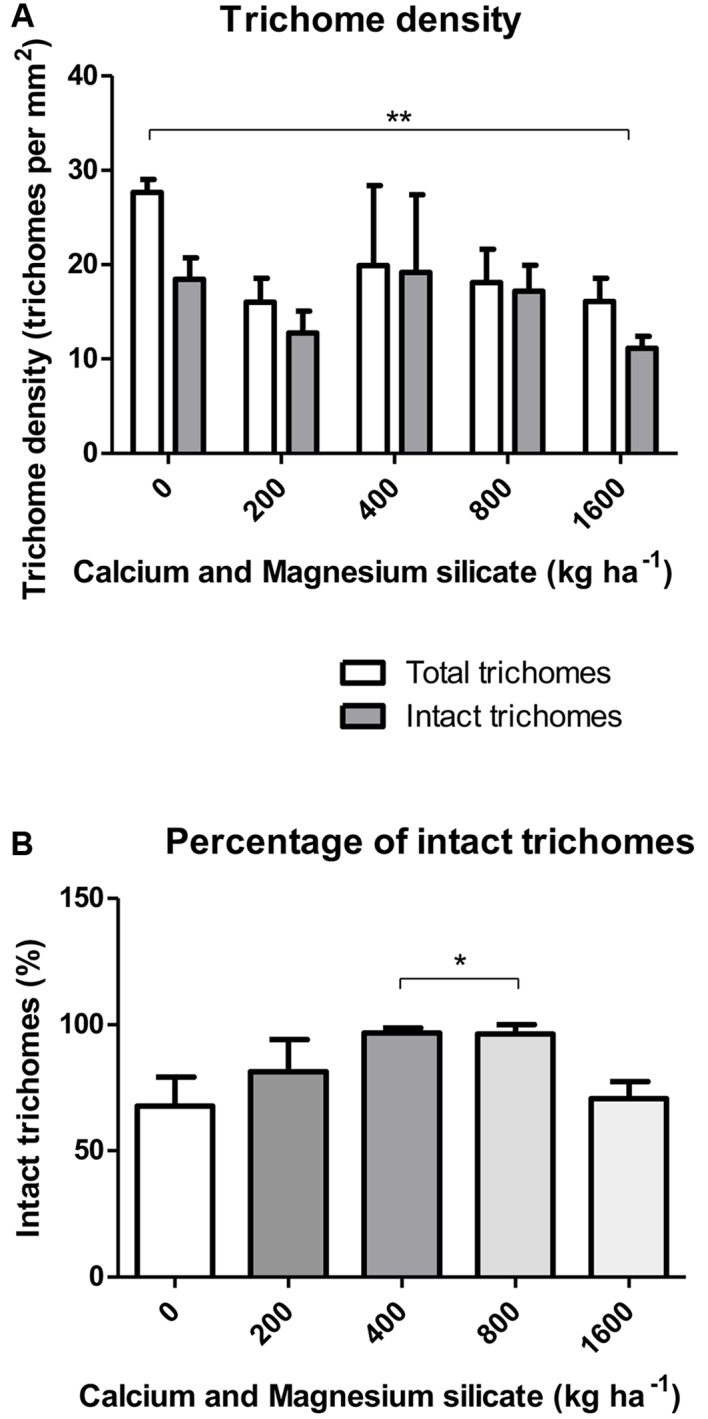
**(A)** Density of total and intact trichomes **(B)** Percentage of intact glandular trichomes in *A. annua* leaves after calcium/magnesium silicate application in the soil. Data represent mean ± MSE. ^∗^*p* < 0.05; ^∗∗^*p* < 0.01.

**FIGURE 3:**
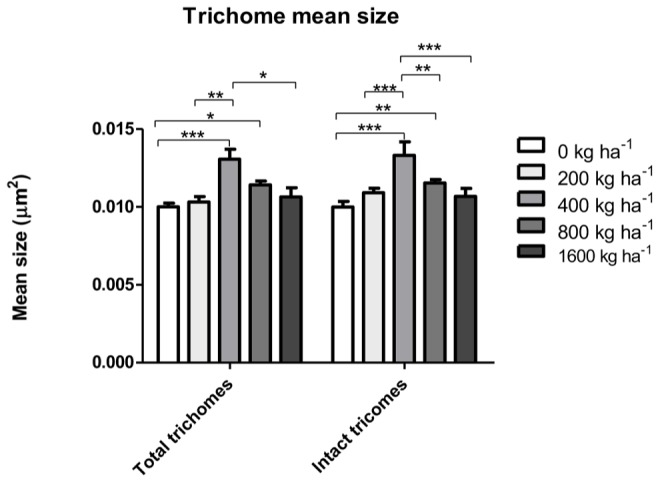
**Total and intact trichome mean sizes in *A. annua* leaves with or without calcium/magnesium silicate applications in the soil.** Data represent mean ± MSE. ^∗^*p* < 0.05; ^∗∗^*p* < 0.01; ^∗∗∗^*p* < 0.001.

**FIGURE 4:**
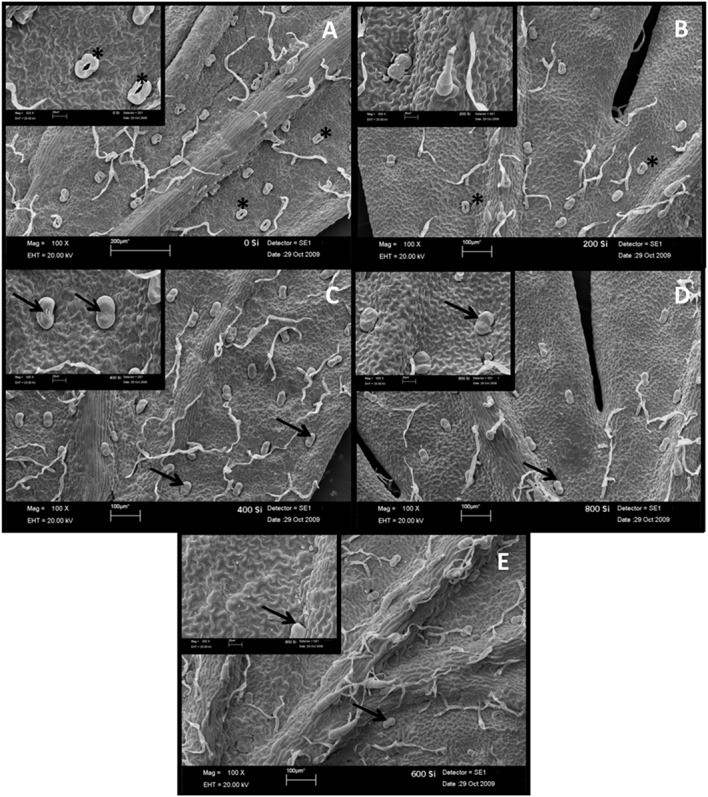
**Scanning electron microscopy of the leaf abaxial surface from *A. annua* grown without (A) or with 200 (B), 400 (C), 800 (D), and 1600 kg ha^-1^ (E) of calcium/magnesium silicate application to the soil.** Arrows indicate intact/not disrupted glandular trichomes and asterisks indicate disrupted glandular trichomes.

The density of total glandular trichomes in *A. annua* did not increase with Si applications, when compared to the control (**Figure 2A**). Indeed, the soil without Si application revealed density mean value of 28.5 ± 1.2 of total trichomes per mm^2^; significantly higher than the values from Si-treated soil (^∗∗^p < 0.01). On the other hand, no significant differences were observed concerning the density of intact trichomes among all tested conditions, i.e., application of 0, 400, and 800 kg ha^-1^ of Si to the soil. In contrast, the percentages of intact glandular trichomes in *A. annua* leaves after Si application in the soil was significantly higher for the plants grown with 400 or 800 kg ha^-1^ of Si, which present around 100% of all intact glandular trichomes (^∗^*p* < 0.05; **Figure 2B**). Furthermore, the Si effects on trichome size were considerably distinct in the 400 and 800 treatments; both increased the sizes of total and intact glandular trichomes, relative to the mean size of total and intact trichomes in the control (**Figure 3**). These findings can be visualized in **Figure 4**, showing the disrupted glandular trichomes for plants without the application of silicate and the number of intact trichomes in plants with 200–1600 kg ha^-1^ of silicate (**Figures 4B–E**), mostly with 400 kg ha^-1^ of silicate (**Figure 4C**). These results indicate that the application of 400 kg ha^-1^ of silicate, corresponding to 32.5 mg kg^-1^ of Si, induced the highest density of intact glandular trichomes. These trichomes probably contained more plant metabolites including artemisinin, compared to the opened or disrupted trichomes, which were mostly found in *A. annua* leaves without the application of silicate.

**FIGURE 5:**
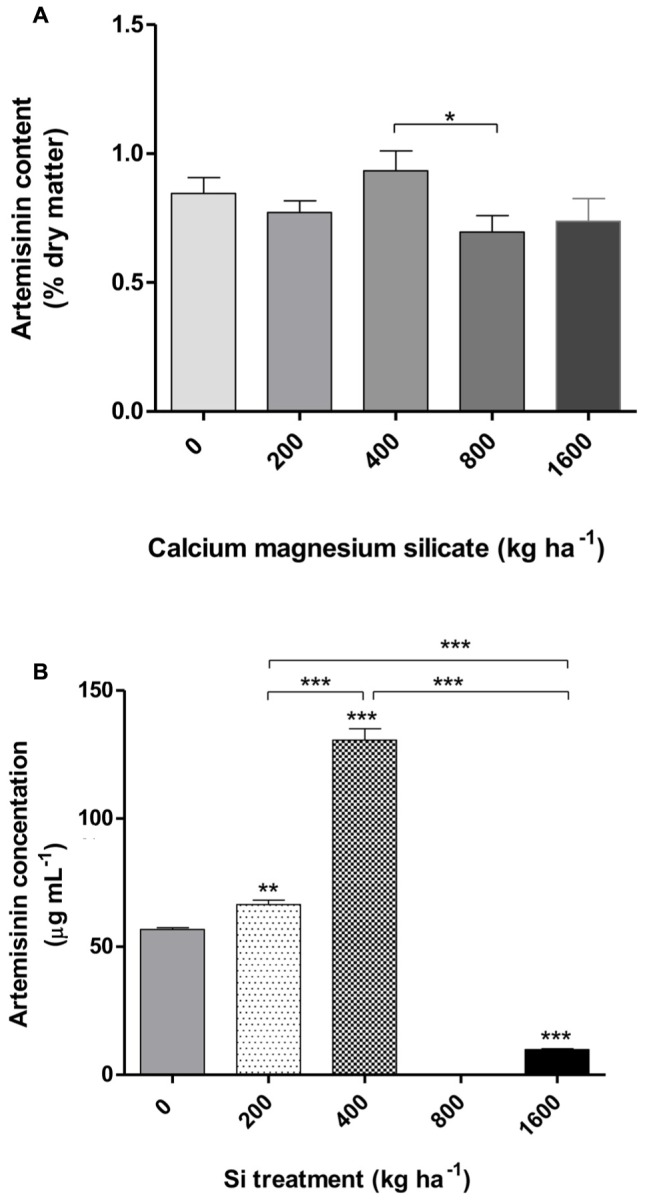
**Artemisinin content in *A. annua* leaves determined by TLC (thin layer chromatography) (A) and plant infusion determined by HPLC (high performance liquid chromatography) (B) obtained after calcium/magnesium silicate application to the soil.** Data represent mean ± MSE. ^∗^*p* < 0.05; ^∗∗^*p* < 0.01; ^∗∗∗^*p* < 0.001 in comparison with the control (0 kg ha^-1^ silicate) or as indicated.

Similar results were obtained when the artemisinin concentrations in *A. annua* leaves were analyzed by TLC (**Figure 5A**; **Table 1**), showing a tendency of higher artemisinin concentration for 400 kg ha^-1^ of silicate, which was significantly higher in comparison with the dose of 800 kg ha^-1^ of silicate. The artemisinin concentration was also evaluated by HPLC in *A. annua* infusion obtained from plants grown under different doses of silicate (**Figure 5B**; **Table 2**). The mean concentration of artemisinin ranged from 9.9 μg mL^-1^ to 130.5 μg mL^-1^, except for the samples obtained with the application of 800 kg ha^-1^ of silicate, in which artemisinin could not be detected. The highest artemisinin concentration (130.5 μg mL^-1^) was detected for the dose of 400 kg ha^-1^ of silicate. Chromatograms displaying the peaks of artemisinin (5.7 min) for each plant infusion sample, obtained from the different silicate dosages applied to the soil, are shown in the **Supplementary Figure S1**.

### Cytotoxicity Assay of *A. annua* Infusion

**FIGURE 6:**
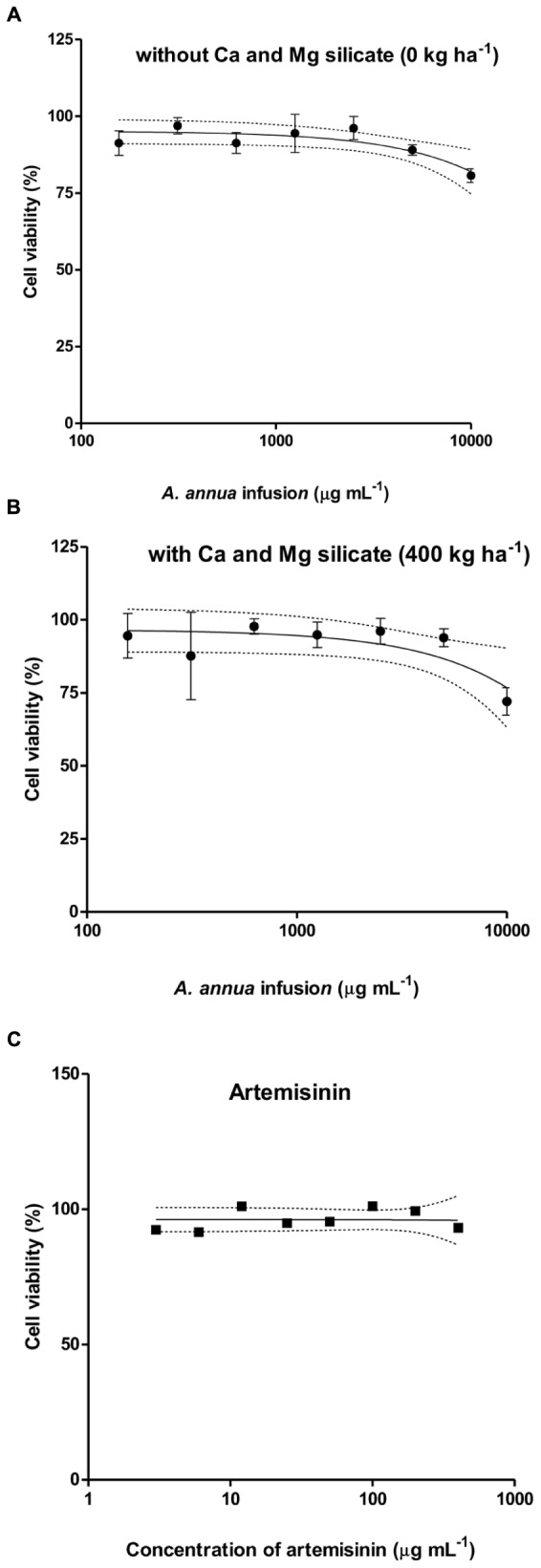
**Cytotoxicity of *A. annua* infusion samples obtained without (A) or with 400 kg ha^-1^ (B) calcium/magnesium silicate applied to the soil, comparatively to pure artemisinin (C), determined by MTT assays.** The results are expressed as percentage of viable cells compared to control.

The cytotoxicity assay to host cells treated with *A. annua* infusion samples obtained from the application of 400 kg ha^-1^ of silicate was selected because this condition showed the highest level of artemisinin concentration. The comparison was carried out for the treatment with infusion samples without silicate application, as well as for pure artemisinin. The following cell viability rates were found: (i) higher than 80% for the plant infusion obtained without silicate application (**Figure 6A**); (ii) higher than 70% for the infusion obtained with the application of 400 kg ha^-1^ silicate (**Figure 6B**); and (iii) higher than 90% for pure artemisinin (**Figure 6C**) and all analyzed concentrations. The plant infusion concentrations ranging from 156 to 2.500 μg mL^-1^ were selected for further *in vitro* experiments of *T. gondii* infection, resulting in higher than 87% cell viability. As control, the artemisinin concentrations ranging from 1.25 to 100 μg mL^-1^ were selected, corresponding to higher than 90% cell viability.

### Effect of Treatment with *A. annua* Infusion on *T. gondii* Growth

**FIGURE 7:**
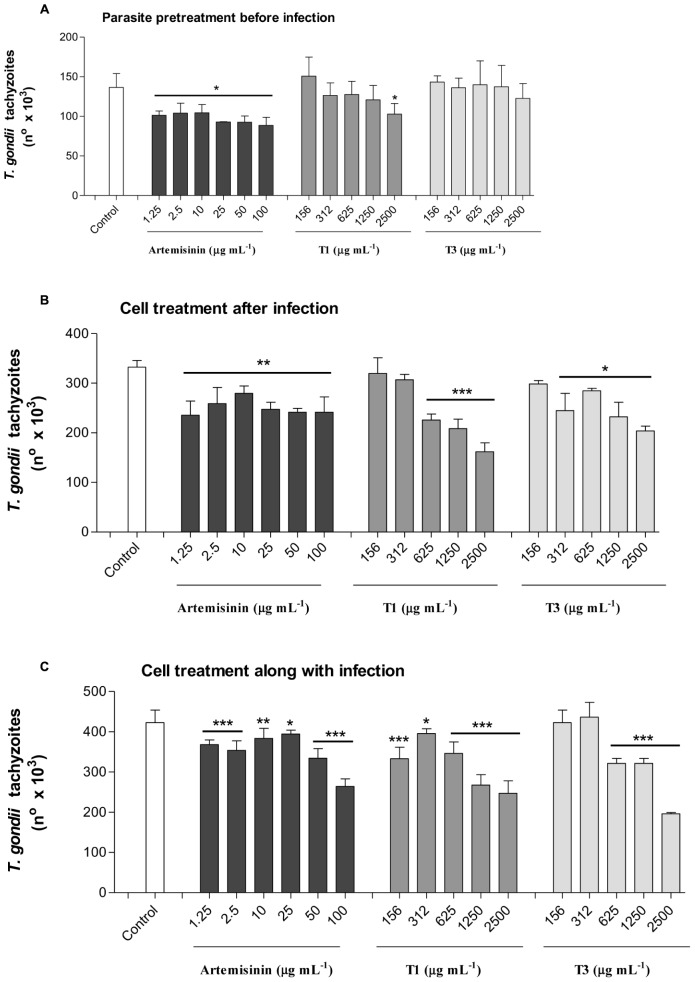
***Toxoplasma gondii* proliferation evaluated by a β-galactosidase colorimetric assay. (A)** Pretreatment of parasites with *A. annua* infusion samples or pure artemisinin before infection; **(B)** Treatment of host cells with *A. annua* infusion samples or pure artemisinin after infection; **(C)** Treatment of host cells with *A. annua* infusion samples or pure artemisinin simultaneously to the infection. Two-fold serial dilutions of *A. annua* infusion (2500 to 156 μg mL^-1^) obtained from plants without (T1) or with (T3) calcium/magnesium silicate applied to the soil (400 kg ha^-1^), compared to pure artemisinin (100 to 1.25 μg mL^-1^) or medium alone (control). Data are expressed as mean ± MSE of the number of tachyzoites calculated in relation to a reference curve. ^∗^*p* < 0.05; ^∗∗^*p* < 0.01; ^∗∗∗^*p* < 0.001.

The effects of *A. annua* infusion samples obtained without or with 400 kg ha^-1^ of silicate, compared to pure artemisinin, on *T. gondii* proliferation were evaluated under three experimental conditions (**Figure 7**). Firstly, we analyzed the effects of the infusion treatment on the parasite before host cell infection (**Figure 7A**). The treatment with plant infusion without silicate application was able to decrease the parasite proliferation only in the highest concentration (2,500 μg mL^-1^), when compared to infected and untreated controls. In contrast, the treatment with plant infusion obtained from the application of 400 kg ha^-1^ of silicate was unable to change the parasite growth at any analyzed concentrations. The treatment with pure artemisinin decreased parasite proliferation at all examined concentrations, although without a dose-dependent response. Secondly, the effects of artemisinin and infusion treatments on cells already infected with *T. gondii* were analyzed (**Figure 7B**). In this case, the results showed that the parasite proliferation was reduced in a dose-dependent manner for both infusions, with and without silicate application, by the concentrations of 312 and 625 μg mL^-1^, respectively, when compared to the infected and untreated controls. Again, artemisinin treatment decreased the parasite growth, although without a dose-dependent response. Finally, the effects of artemisinin and infusion treatments on cell along with the infection were investigated (**Figure 7C**). Here, all the concentrations of the plant infusion obtained without silicate application were able to decrease parasite replication compared to the infected and untreated controls, but with a dose-dependent response from 312 μg mL^-1^ onward. For the plant infusion obtained from 400 kg ha^-1^ of silicate application, there was a dose-dependent reduction from 625 μg mL^-1^ onward. Artemisinin also decreased the parasite growth in a dose-dependent manner for all analyzed concentrations.

## Discussion

Silicon is an abundant soil element but not all Si forms present in the soil are available for plant uptake. Usually, Si is found as silicate minerals, aluminum silicates, and various forms of silicon dioxide (SiO_2_). Used as a plant nutrient, Si fertilization improves crop productivity and plant health (Guntzer et al., 2012; Haynes, 2014).

Plants absorb Si in the form of monosilicic acid (H_4_SiO_4_), which is found in both liquid and adsorbed phases of Si in soils. The monosilicic acid behaves as a very weak acid, and even in pH 7.0, only 2 mg kg^-1^ are ionized (H_3_SiO_4_^-^); as pH increases the degree of ionization increases as well. Its concentration in soil solution varies according to clay content, iron and aluminum oxides or hydroxides, organic matter, and pH. Soil pH can be corrected with liming materials, modifying the solubility of the monosilicic acid. This fact is important for rice, once maximum silicon absorption occurs within the 4.7–7.6 pH range (Guntzer et al., 2012; Tubaña and Heckman, 2015).

Some soils as Oxisols (used in the research) and Ultisols demonstrate low levels of Si available to the plants in their native state. These soils are leached, acid, highly weathered, and low in base saturation (Foy, 1992). Thus, Ca and Mg silicate fertilization can improve fertility of such soils. Si can enhance the growth and development of several plant species including rice, sugarcane, most other cereals, and several dicotyledons (Jones and Handreck, 1967; Elawad and Green, 1979; Bélanger et al., 1995; Savant et al., 1997). In addition, Si amendments proved effective in controlling several important plant diseases. Nevertheless, in this research work soil amended with different Si doses demonstrated significant positive correlation only with N at the dose of 1600 kg ha^-1^(**Table 3**). There appeared to be a silicon-induced increase in leaf nitrogen concentration, but statistically significant only at the highest dose. In this study, urea and soil organic matter were the possible nitrogen sources. Therefore, this correlation remains to be explored. Nitrogen is an important component of amino acids, proteins, nucleic acids, and many other compounds. Other foliar macronutrients were not significantly affected by Si treatments. The role of macronutrients in *A*. *annua* leaves have already been investigated by Ferreira (2007) who showed that the nutrient least required for biomass accumulation per plant was K, followed by P, and N, and that the artemisinin concentration was significantly higher in -K plants when compared to plants under the complete treatment.

**Table 3:** Macronutients present in the leaves of *A. annua* grown in soil treated with Si at doses of 0, 200, 400, 800, or 1600 kg ha^-1^.

Mean average macronutrient status (g kg^-1^)					
Macronutrients	Calcium and Magnesium silicate (kg ha^-1^)				
	0	200	400	800	1600
N	19.1	17.0	17.7	19.5	29.5
P	1.4	1.4	1.4	1.6	1.6
K	16.7	17.3	16.4	17.3	18.0
Ca	11.8	12.7	13.4	13.1	13.3
Mg	3.9	4.2	5.2	4.1	4.3
S	5.4	5.0	5.0	5.3	4.6

Although statistically insignificant, Si treatments increased the average foliar Si content by 0.1%, from 0.3% for 0 kg ha^-1^ to 0.4% for 1600 kg ha^-1^. According to criteria established by Ma and Takahashi (2002), plants with a Si content and Si/Ca ratio higher than 1.0% and 1.0, respectively, are defined as silicon accumulators. By contrast, plants with a Si content lower than 0.5% and Si/Ca ratio lower than 0.5 are defined as Si non-accumulating plants. For monocotyledons species, the Si content is high and the Ca content is low. For dicotyledon species, occurs the opposite. In this study, the Si/Ca ratio was 0.25 and 0.3 for 0 kg ha^-1^ and 1600 kg ha^-1^ treatments, respectively. Further, according to Mitani and Ma (2005) the low accumulation of Si in dicots is due to a lack of specific transporters to facilitate the radial transport and the xylem loading of Si and suggested that the transport of silicon across cells was accomplished via a passive diffusion mechanism. Thus, the analysis of foliar mineral composition indicates that *A. annua* is a Si non-accumulating plant and does not have an active Si-uptake mechanism. Still, Si application in our experiment was positively correlated with artemisinin content and trichome integrity perhaps due to the fact that the initial soil demonstrated low pH and low Si content.

Micronutrient status was also not significantly altered by any silicate doses applied to the soil (**Table 4**). Therefore, suggesting that *A*. *annua* plants had sufficient amounts of micronutrients for the growth and production of artemisinin. Previous studies evaluating the micronutrient content in *A*. *annua*, demonstrated that the amount of artemisinin decreased in the absence of copper (Cu) and especially of boron (B; Srivastava and Sharma, 1990). Moreover, in another research work, B-deficient plants did not bloom, leading to a reduction of artemisinin content by 50% (Brisibe et al., 2008).

**Table 4:** Micronutrients present in the leaves of *A. annua* grown in soil treated with Si at doses of 0, 200, 400, 800, or 1600 kg ha^-1^.

Mean average micronutrient status (g kg^-1^)					
Micronutrients	Calcium and Magnesium silicate (kg ha^-^^1^)				
	0	200	400	800	1600
B	19.1	17.0	17.7	19.5	29.5
Mn	1.4	1.4	1.4	1.6	1.6
Cu	16.7	17.3	16.4	17.3	18.0
Fe	11.8	12.7	13.4	13.1	13.3
Zn	3.9	4.2	5.2	4.1	4.3

The effects of Si on plant growth and productivity have already been investigated (Datnoff et al., 2001; Cooke and DeGabriel, 2016 and Coskun et al., 2016; Reynolds et al., 2016), but to date there is no information regarding Si effect on *A. annua* and the development the glandular trichome. Further, there is limited data regarding the use of *A. annua* infusion on *T. gondii* infection. In the present study, we investigated the response *A. annua* to Si signal, considering the content of the active principle, the artemisinin, in the inhibition of *T. gondii* infection. Four different Si doses and three different infusion assays were assessed. The 400 and 800 kg ha^-1^ Si doses notably increased the mean size of the intact glandular trichomes.

Artemisinin is stored in glandular trichomes in the aerial parts of *A. annua* (Duke and Paul, 1993). Trichomes had already been identified by SEM and X-ray microanalysis, showing the deposition of Si in the cell wall of wheat leaves and the location of silica acting as a physical barrier (Epstein, 2009). In this study, the effects of Si on the artemisinin content were investigated by analysis of trichomes and it was found that, even though the mean density of total glandular trichomes was higher without Si application, the percentages of intact glandular trichomes in *A. annua* leaves after Si applications to the soil was significantly higher for the plants grown with 400 or 800 kg ha^-1^ Si, which present about 100% of all intact glandular trichomes.

Artemisinin solubility was not appropriate for the analysis of the samples by TLC in the present study, since the artemisinin concentration was determined on dried leaves of *A. annua* and expressed as percentage of dry weight. Also, samples obtained from the treatment with 800 kg ha^-1^ of silicate showed lower artemisinin concentration compared to the treatment with400 kg ha^-1^ of silicate in the soil. However, using the HPLC it was possible to detect high concentrations of artemisinin in *A. annua* infusions, even in the control condition, when the plant was cultivated without the application of silicate (56.7 μg mL^-1^ of artemisinin). These results are higher than those found by Mueller et al. (2000) (24.5 μg mL^-1^) and Jansen (2006) (24.2 μg mL^-1^), using similar infusion preparations and analysis methods. Furthermore, the dose of 400 kg ha^-1^ of silicate induced higher concentrations of artemisinin than those previously reported in plants without the application of silicate (94.0 μg mL^-1^; Mueller et al., 2004; Räth et al., 2004). Clearly, these results show wide variation in the artemisinin content in *A. annua* infusion samples, indicating that many factors, such as temperature, contact time, percentage of material, solvent, and plant genetics can influence the solubility of artemisinin and its subsequent therapeutic effects (van der Kooy and Verpoorte, 2011; Rocha e Silva et al., 2012). In this context, it is likely that the unexpected very low concentrations of artemisinin in infusion samples, which were obtained from *A. annua* treated with 1600 kg ha^-1^ and, especially with 800 kg ha^-1^ of silicate, may have been affected by some of these factors, which may impact the solubility of artemisinin, resulting in almost undetectable levels.

To date, the infusion of *A. annua* is not considered as an option for the treatment of toxoplasmosis. Currently, conventional drugs, as sulfadiazine/pyrimethamine, are the first choice for treatment of the clinical presentations of toxoplasmosis (Petersen and Schmidt, 2003). Nevertheless, due to the severe side effects, including bone marrow suppression, this conventional procedure demands the concomitant administration of folinic acid to alleviate these effects (Martins-Duarte et al., 2006). Furthermore, this treatment is frequently not well tolerated, leading to the substitution of sulfadiazine by other drugs such as clindamycin (Montoya and Liesenfeld, 2004). These major concerns have stimulated the research toward an alternative and less toxic drug against *T. gondii* infection.

Although *A. annua* infusion is not commonly used to control toxoplasmosis, as it is for malaria, in the present study the effect on *T. gondii* intracellular proliferation was tested as an alternative therapy, since its ability to decrease the tachyzoite replication may be useful in the treatment of acute primary toxoplasmosis or reactivation of chronic infection. However, even though multiple phenolic constituents, as chlorogenic acids, play a role in enhancing artemisinin solubility and extractability in *A. annua* aqueous preparations (Carbonara et al., 2012), the low solubility of artemisinin when compared to pure artemisinin, which is soluble in distilled water and demonstrates fair stability in neutral solvents, still handicaps the usefulness of *A. annua* therapeutic infusion.

Considering all the results obtained from the *A. annua* infusion samples in the present study, there was a decrease in parasite proliferation achieved with lower doses of plant infusions obtained from the application of silicate. Still, the infusion obtained from Si treatments was as effective as the infusion obtained from –Si treatments, even though the concentration of artemisinin was higher in *A. annua* treated with 400 kg ha^-1^ of silicate. On the other hand, parasite pretreatment with infusion samples before infection was less effective in controlling parasite proliferation for both samples, with or without silicate application. As expected, the pure artemisinin was able to decrease parasite proliferation in all experimental conditions, as previously reported.

Jones-Brando et al. (2006) and Hencken et al. (2010) investigated four artemisinin derivatives, which showed an increase in anti-*Toxoplasma* activity and decreased cytotoxicity as compared to trimethoprim. Unsaturated synthetic derivatives, known as carba artemisinin, were also able to inhibit various stages of *T. gondii*, emerging as therapeutic agents for the prevention and treatment of toxoplasmosis in humans (D’Angelo et al., 2009).

In malaria parasite, the artemisinin triggers the inhibition of calcium-ATPase of the endoplasmic reticulum sarcoplasm (SERCA; Woodrow et al., 2005). Nagamune et al. (2007) evaluated chemically derived mutants of *T. gondii* resistant to artemisinin and concluded that the resistance was not due to molecular changes in the SERCA expression, but in the induction of microneme secretion, a calcium-dependent process that is triggered by artemisinin. These studies suggest a calcium homeostasis in the mechanism of action of artemisinin against parasites from Apicomplexa phylum. In the present study, however, it was observed no variation in Ca content in leaves treated with any Si concentration, when compared with untreated plant, excluding the role of this macronutrient in the results obtained in our experimental design.

The use of *A. annua* infusion in *T. gondii* infection was demonstrated for the first time in our previous study, showing that the plant infusion can be a potential therapeutic alternative to control the growth of the parasite in both *in vitro* and *in vivo* experimental conditions (Oliveira et al., 2009). In the present study, the application of silicate in the soil had a positive effect on the size of glandular trichomes and hence on the artemisinin content, but this result was not associated with enhanced efficacy of leaf infusion from *A. annua* grown in the soil with silicate application on *T. gondii* replication. These data suggest that other components in addition to artemisinin could contribute to the effect of the plant infusion. In this context, it is known that *A. annua* infusion is rich in phenolic antioxidants, especially flavonoids (Räth et al., 2004). As the artemisinin content in *A. annua* leaves is approximately 1%, it is questionable whether all the benefits of the traditional tea are uniquely assigned to artemisinin (Ferreira et al., 2010). Among the advantages of using the *A. annua* infusion, there is the possibility of producing and preparing plant medication without the need for purification, which causes the loss of other valuable compounds from plants, such as flavonoids, flavones, and coumarins.

## Conclusion

The application of Si to the soil at concentration of 400 kg ha^-1^ had beneficial effects on the *A. annua* physiology, such as the size of total and intact glandular trichomes, which increased the capability of storage and content of artemisinin in *A. annua* leaves and infusion. The opposite effect, however, was observed when higher concentrations of Si, i.e., 800 or 1600 kg ha^-1^ of silicate were applied to the soil, resulting in almost undetectable levels of artemisinin in the *A. annua* infusion. Thus, further studies are necessary to investigate the extractability of artemisinin from leaves containing high-size trichomes. Also, considering that the Si treatment in the soil had little influence on the effect of *A. annua* infusion to control *T. gondii* growth, since both types of infusions (with or without the application of silicate) were able to reduce parasite proliferation, other components present in the leaves may be acting in synergy with the artemisinin, and should be investigated.

## Author Contributions

CR, CM, and TO carried out the majority of the experiments with *A. annua*, from the plant cultivation to the extract assays in cell cultures. FS carried out the experiments related to the parasite maintenance in cell culture and the HPLC analysis. LO, GK, and RL performed the experiments concerning the analysis of macro and micronutrients, as well as the Si content in *A. annua*. MR and NN participated in the surface analysis of the glandular trichomes in *A. annua* leaves by scanning electron microscopy. XS performed the experiments to determine the artemisinin content in *A. annua* leaves by thin layer chromatography. CR, TM, DS, and JM participated in the experimental design throughout this research, as well as in the data analysis and revision of the manuscript.

## Conflict of Interest Statement

The authors declare that the research was conducted in the absence of any commercial or financial relationships that could be construed as a potential conflict of interest.

## Supplementary material

FIGURE S1 **High performance liquid chromatography (HPLC) chromatograms determined for *Artemisia annua* infusion samples obtained after calcium/magnesium silicate application to the soil at different concentrations, as follow: (A) 0 kg ha^-1^; (B) 200 kg ha^-1^; (C) 400 kg ha^-1^; (D) 800 kg ha^-1^; (E) 1600 kg ha^-1^.** Arrows indicate the peak of artemisinin (5.7 min) according to the reference curve.
