# Supplementary figures and images for: Si-Accumulation In Artemisia annua Glandular Trichomes Increases Artemisinin Concentration, but Does Not Interfere In the Impairment of Toxoplasma gondii Growth
Source: Front Plant Sci. 2016 Sep 23;7:1430. doi: 10.3389/fpls.2016.01430 (PMC5033981; doi:10.3389/fpls.2016.01430)

Supplementary Figure S1

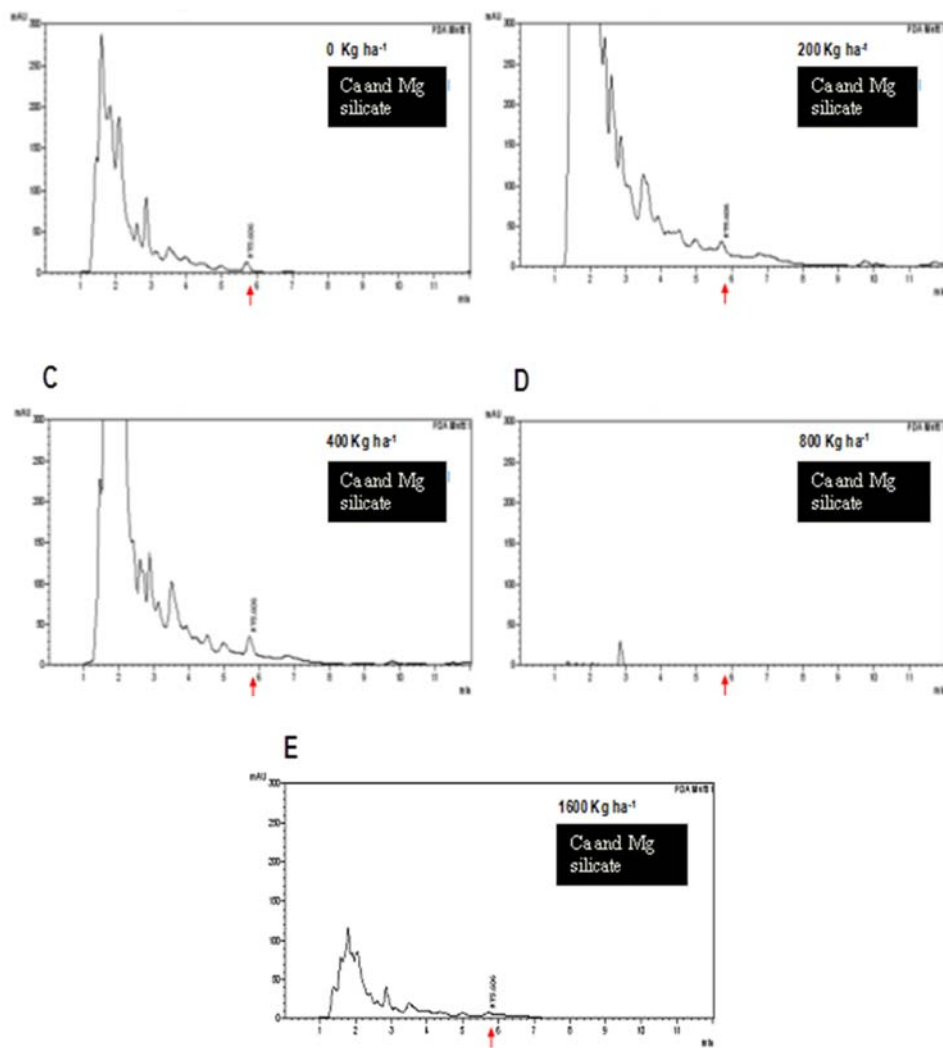

Supplement: FIGURE S1 — High performance liquid chromatography (HPLC) chromatograms determined for Artemisia annua infusion samples obtained after calcium/magnesium silicate application to the soil at different concentrations, as follow: (A) 0 kg ha-1; (B) 200 kg ha-1; (C) 400 kg ha-1; (D) 800 kg ha-1; (E) 1600 kg ha-1. Arrows indicate the peak of artemisinin (5.7 min) according to the reference curve. [file Image_1.PDF]
